# Role of Immunotherapy in the Treatment of Cancer: A Systematic Review

**DOI:** 10.3390/cancers14215205

**Published:** 2022-10-24

**Authors:** Sia Pei Ling, Long Chiau Ming, Jagjit Singh Dhaliwal, Madhu Gupta, Chrismawan Ardianto, Khang Wen Goh, Zahid Hussain, Naeem Shafqat

**Affiliations:** 1 PAPRSB Institute of Health Sciences, Universiti Brunei Darussalam, Gadong BE1410, Brunei; 2 Department of Pharmacy Practice, Faculty of Pharmacy, Universitas Airlangga, Surabaya 60115, Indonesia; 3 School of Pharmaceutical Sciences, Delhi Pharmaceutical Sciences and Research University (DPSRU), New Delhi 110017, India; 4 Faculty of Data Science and Information Technology, INTI International University, Nilai 71800, Malaysia; 5 Faculty of Health, University of Canberra, Bruce, ACT 2617, Australia

**Keywords:** cancer cell, breast cancer, neoplasm, non-small cell lung cancer, glioblastoma, antineoplastic agent, preventable death, medicine, biological therapy, immunomodulation

## Abstract

**Simple Summary:**

The main purpose of this article is to review the efficacy of immunotherapy either as a stand-alone treatment or in combination with the available conventional cancer treatment in stopping the reoccurrence of cancer. The article will assess and determine the efficacy of immunotherapy in the treatment of cancer via the overall survival and progression-free survival rate.

**Abstract:**

Tremendous progress has been made in cancer research over the years, and, as a result, immunotherapy has emerged as an important therapy for the treatment of cancer, either as a stand-alone treatment or in conjunction with other cancer therapies. Immunotherapy has demonstrated encouraging outcomes and offers a viable strategy for not only enhancing the quality of life but also dramatically boosting the overall survival rate of cancer patients. The objective of this systematic review was to assess the efficacy of immunotherapy in the treatment of cancer. Databases such as PubMed and Science Direct were searched from their inception until September 2021, using the following keywords: cancer immunotherapy, cancer recurrence, cancer treatment options, and cancer therapies. The systematic review was conducted in accordance with the PRISMA protocol. There were a total of 599 articles; however, after applying the inclusion and exclusion criteria, the final review ended up with 34 publications. In conclusion, the studies have demonstrated that immunotherapy is a viable alternative treatment option for patients with recurrent or metastatic cancer, since the overall survival rate and progression-free survival rate were shown to be successful.

## 1. Introduction

Cancer is considered as the second-leading cause of mortality in global map after cardiovascular disease. According to recent data from GLOBOCAN 2020, there were an estimated 10 million deaths worldwide caused by cancer in the year 2020 alone [1]. Among the different types of cancer, breast cancer is the most commonly diagnosed cancer worldwide (2 million cases), followed by lung, colorectum, prostate, skin (non-melanoma), and stomach cancer, respectively. In addition, it has been anticipated that there will be a tremendous increase in the elderly population around the world, leading to a cohort of elderly people with a higher risk of getting cancer due to age-related health deterioration [2].

Nevertheless, there have been major technological advances in cancer treatment during the last century, despite its inevitable side effects and the inadequacy it may bring upon treatment [3]. In fact, prior to the start of suitable cancer treatment, the patients who are diagnosed in an early stage of the disease showed a significant trend toward the overall survival rate and were offered a cost-effective means of cancer treatment, as compared to those diagnosed at a later stage [4]. The main purpose of a treatment regimen is to cure cancer and to prolong the patient’s life span by slowing down or blocking the growth of cancer cells. However, the treatment of cancer may vary depending on an early or late diagnosis, which will determine whether it has metastasized or not.

Over the years, surgery, radiotherapy, and chemotherapy have been considered as the three main pillars in cancer treatment, but, with the success in using immune treatment either alone or in combination with other cancer therapies, immunotherapy has emerged as the fourth crucial pillar in combating the disease [4]. Unlike other cancer treatments, immunotherapy utilizes the body’s own immune system to recognize and attack cancer cells and, hence, offers a natural approach in controlling the progression of the disease. Most cancer therapies involving either surgery, radiotherapy, or chemotherapy have shown to be effective in the treatment of primary tumors, but relapse of the disease is still a typical recurring issue due to the presence of remaining malignant cells or tumor metastases [5]. Therefore, immunotherapy serves as one of the alternative or additional approaches, which utilizes the immune checkpoint inhibitors, chimeric antigen receptor (CAR) T-cell therapy, and cancer vaccines for the treatment of cancer [6,7].

Overall, the main purpose of this article is to review the efficacy of immunotherapy either as a stand-alone treatment or in combination with the available conventional cancer treatment in stopping the reoccurrence of cancer.

## 2. Methods

### 2.1. Search Strategy

The appropriate keywords, such as cancer immunotherapy, cancer recurrence, cancer treatment options, and cancer therapies were used to search in the PubMed and ScienceDirect databases for research articles published from their inception to September 2021. The language used to search for the research articles was limited to English only.

### 2.2. Eligibility Criteria

Study designs such as randomized controlled trials, non-randomized clinical trials, and prospective studies were included to further assess the efficacy of immunotherapy either as a stand-alone treatment or in combination with any of the typical cancer treatments used. However, research articles must mention the use of immunotherapy in patients with ongoing cancer treatment or cancer recurrence, to evaluate the treatment’s efficacy in prolonging the overall cancer survival rate. Apart from that, any articles that include pre-clinical study, case reports or series, retrospective study, systematic review, or meta-analysis were also excluded.

### 2.3. Selection and Data Collection Process

The articles were thoroughly reviewed in order to select those articles that had fulfilled all the requirements established for the synthesis of this systematic review. The acquired data were then subsequently assessed and compiled by the authors.

### 2.4. Risk of Bias Assessment

The Cochrane assessment tool was used to assess the risk of bias and methodological quality in the included studies. The ROBINS-I tool was used to assess risk of bias in the results of the non-randomized studies included [8]. As for the randomized studies, the Cochrane risk-of-bias (RoB 2) tool was used instead [9]. Both of these tools require judgment, on the risk of bias arising from each domain, by answering the signaling questions. The overall judgment will result in the overall risk of bias.

### 2.5. Data Analysis

The following information from each of the eligible studies were extracted, which were according to the name of the study, first author and year of publication, study design, study phase, type of cancer, number of patients, mean age, treatment groups, and the patients’ overall survival and progression-free survival rates.

## 3. Results

### 3.1. Study Selection

**Figure 1:**
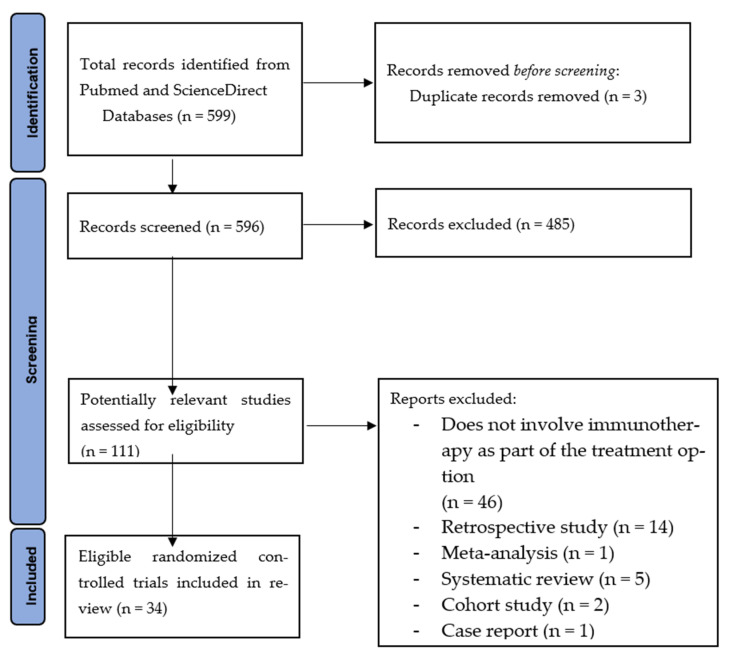
PRISMA flow chart.

A total of 599 articles were found prior to the database search, but only 111 potentially relevant articles were selected after the full-text screening. After a comprehensive review of the selected articles, 34 articles of both non-randomized trial and randomized controlled trials, fulfilling the inclusion criteria, were selected. The PRISMA flow chart is presented in Figure 1. Any articles that did not meet the inclusion criteria were excluded from this study, because they did not provide any information regarding the main objective of this review. Studies with human subjects were particularly chosen as part of the criteria, instead of animal studies, as the data from the patients would give an overall outcome of the interventions used. There were 22 non-randomized trials and 12 randomized controlled trials, which include the use of PD-1 inhibitors, vaccines, anti-EpCAM and anti-CD3 monoclonal antibodies, CTLA-4 antagonist, adoptive cell therapy, CD22-specific conjugated antibody, and antineoplastics. The 34 selected studies include 5 phase 1 trials, 9 phase 1–2 trials, 11 phase 2 trials, 1 phase 2–3 trials, and 8 phase 3 trials. These trial studies were grouped accordingly to the type of cancers, as shown in Table 1. Median overall survival and progression-free survival were assessed for each study, as they were the primary results used for this review.

### 3.2. Reporting Biases

There were a few confounding factors identified for the risk of bias for the non-randomized controlled trials in Table 2(A). One of the factors is hormonal therapy, which was seen in prostate cancer patients receiving luteinizing hormone-releasing hormone (LHRH) analogs during the treatment [32] Patients receiving this additional treatment along with immunotherapy may have had an influence on the overall effect of the results, as LHRH analogs aid in the inhibition of prostate cancer growth [44]. The other confounding factor were in patients with non-small cell lung cancer who are current smokers [16]. The results of the immunotherapy used in this case may be affected, as patients who are current smokers may reduce the efficacy of the treatment [45]. Hence, the confounding factors mentioned above may lead to distortion of the actual results in the efficacy of immunotherapy in cancer treatment.

All of the non-randomized trial studies included had a ‘moderate’ bias in the measurement of the outcome, due to the fact that the majority of the trials were open-label, which meant that the assessors were aware of the intervention received by the study participants.

As for the risk of bias in the randomized controlled trials in Table 2(B), most of the studies included had ‘some concerns’ in the bias arising from the randomization process and due to deviations from the intended interventions. Three studies, including Schmid et al. [18], Shore et al. [11], and Liau et al. [21], did not have any information on the type of randomization methods used or the interventions used on the participants, which raises concerns regarding the randomization process. In addition, most of the included studies were open-label studies even though they were randomized, except for Harper et al. [24] and Hemstock et al. [36].

## 4. Discussion

The results from the present study indicate that the use of immunotherapy, either alone or as a supportive therapy to the conventional cancer treatments, has enormous potential in improving the overall survival and progression-free survival rates of cancer patients, especially those who have failed on their first-line therapy, leading to disease recurrence. In addition, the results of the clinical trials have shown a minimal tolerable side effect of the immunotherapy used, unlike the usual treatment such as chemotherapy, whereby there is a higher prevalence of adverse effects, especially among elderly patients [29].

The use of immune checkpoint inhibitors showed a relatively improved response and survival rates of patients with high expression of PD-L1 on their tumor cells, especially in patients with non-small cell lung cancer. Biomarkers, such as PD-L1 and tumor infiltrating immune cells, and genetic mutations are important in cancer, as they help determine what may be the possible cause of the cancer to recur and metastasize. The PD-1 and PD-L1 pathways are rather important for the immune checkpoint inhibitors, as most cancer cells express PD-L1 as cell surface receptors, which play a major role in regulating T-cell exhaustion by binding onto PD-1 [46]. Therefore, targeting the PD-L1 pathway by immune checkpoint inhibitors will block the PD-L1 binding and enhance the immune response against cancer cells. However, despite the immense response seen in PD-L1 positive patients, there have been anti-tumor responses as well, in patients with low or negative PD-L1 expression, from using immune checkpoint inhibitors [13,14].

Additionally, cancer vaccines have shown improvements in the overall results of the studies, as seen in Table 1. The tumor burden elicits an immunosuppressive effect in a recurrent or metastatic cancer environment. Hence, a further approach has been completed to extend the response of the vaccines, such as including the influence of cytokines on the immune response or in combination with antibodies in the inhibition of the receptors, such as CTLA-4 and PD-1, used in downregulating the immune responses [17]. Overall, the main role of these cancer vaccines is to stimulate the immune responses and, thereby, reduce the disease process from either recurring or as a form of prophylaxis of cancer caused by infections.

Although there were effective treatments such as surgery, there have been cases of recurrences and their association with reproductive morbidities. Therefore, cancer vaccines were made to prevent cancer associated with human papillomavirus (HPV) without the need of surgery. One of the clinical trials involved the use of these vaccines in HPV patients associated with cervical intraepithelial neoplasia (CIN) grades 2 or 3. The study showed a reduction in the abnormal cells in the viral DNA, regardless of the high-risk HPV types [24].

Immunotherapy using dendritic cell (DC)-based vaccination has been used in an attempt to treat patients with recurrences after failing their first-line therapy. In cases of sarcoma, further treating these patients with chemotherapy would be insufficient due to the tumors being resistant to the treatment and the rise of multiorgan failure from the treatment. Although there are other possible treatments available, the results are inadequate. Thus, DC-based vaccination offers a much safer treatment, with fewer side effects. As seen in this study, DC-based vaccination has shown to increase the immune responses through the production of IFN-γ and IL-12 [46]. Besides that, the use of DC-based vaccination provided a longer overall survival rate in the treatment of acute myeloid leukemia patients, to further prevent or delay the disease recurrence. The use of this vaccine in the treatment of leukemia is an effective approach toward patients who were unable to carry out an allogeneic hematopoietic stem cell transplant, especially in elderly patients and also in younger patients who may not have compatible donors [37].

Lastly, the use of CAR T-cells therapy-based studies were not as effective as the other two, but they still had an effect on the overall survival rate. Since tumors are often resistant to standard treatment, CAR T-cells have shown some favorable results, especially in CD19-positive malignancies’ clinical trials [26]. CD19 is a biomarker that is critically involved in the malignant tumors of the B-lymphocyte system. CARs bind onto antigens, which are expressed on the cell membrane of tumor cells, and there are a few possible CAR target antigens identified in the case of sarcoma that include human epidermal growth factor receptor 2 (HER2). As there are many types of sarcomas, there happen to be some malignancies of the sarcomas that express low levels of HER2, e.g., osteosarcoma, which may not be so effective for HER2 monoclonal bodies to exert their effect. Overall, HER2 CAR T-cells did demonstrate antitumor activity in patients expressing low levels of HER2. Although the results were for HER2-negative patients, using CAR T-cells targeting HER2 could also be possible in malignancies that are HER2-positive, which have no effects on HER2 antibodies because they are not HER2-gene-amplified [26].

While the results from this systematic review have shown to be promising, there are still numerous ongoing clinical trials that have been performed using immunotherapy in the treatment of cancer. In fact, throughout the years, there have been several immunotherapy drugs that have been approved by the US Food and Drug Administration (FDA) for use in the treatment of a wide range of cancers. According to Benjamin et al. (2022), 42% of the cancer drugs approved by the US FDA between the 1 May 2016 and 31 May 2021 are used in combination with standard therapies or used as an adjuvant or maintenance treatment. Pembrolizumab, which is one of the approved cancer drugs, was used in the treatment of advanced non-small cell lung cancer (NSCLC) alongside chemotherapy, as a combination treatment [47]. Other approved uses of pembrolizumab includes the treatment of head and neck squamous cell carcinoma, gastrointestinal cancer, Hodgkin’s lymphoma, melanoma, and bladder cancer, as an alternative source of treatment when the disease has progressed after standard treatment or where standard treatment is not appropriate enough to be carried out [48].

Also, durvalumab was approved as a maintenance treatment used in patients with unresectable stage 3 NSCLC whose disease remained stagnant after receiving simultaneous platinum-based chemotherapy and radiation therapy [47]. Cemiplimab was approved as a source of alternative treatment in patients with metastatic cutaneious squamous cell carcinoma (CSCC) or locally advanced CSCC who are unable to have curative surgery or radiation [49].

Besides immune checkpoint inhibitors, the US FDA approved CAR T-cell therapy such as tisagenlecleucel and axicabtagene ciloleucel to be used in the treatment of hematological malignancies, acute lymphoblastic leukemia (ALL), and large B-cell lymphomas, particularly in patients whose disease has relapsed and remained refractory despite multiple treatments [50]. In addition, tisagenlecleucel is used in the treatment of pediatric patients with ALL who had a history of refractory disease, though this disease is more commonly diagnosed in children compared to adults [50]. This helped overall in the remission of the disease among pediatric patients where standard treatment is not efficient enough to suppress and prevent the disease.

Regardless of how the FDA approved the use of immunotherapy, either as an alternative or adjuvant cancer therapy, there are immunotherapies that are used as the first line of treatment against cancer. Such examples include the use of pembrolizumab as the first line of treatment in patients with microsatellite instability-high (MSI-H) or mismatch repair-deficient (dMMR) colorectal cancer that has metastasized [51]. Other uses of pembrolizumab as the first line of treatment include either as a monotherapy or in combination with chemotherapy in the treatment of patients with advanced NSCLC [52].

However, despite the promising results that immunotherapy may provide using the body’s immune system to treat a broad range of malignancies, stimulating the immune system may lead to autoimmune toxicity, also known as an immune-related adverse event (irAE). An irAE will occur in about one in five patients receiving immunotherapy, and the risk increases with patients who are concurrently taking two immunotherapy drugs and have had a history of autoimmune disease [53]. The severity of these adverse events (AEs) ranges from mild to life-threatening and is influenced by the type of immunotherapy used, its route of administration, and the mechanism of action [54]. Compared with the AEs of standard chemotherapy, they have a much more predictable nadir or cyclic pattern after administration [54]. In contrast, immunotherapy’s AEs are rather complicated, as they vary in onset and resolution, are present during the first few weeks of administration, and may linger up to a few months after treatment [54].

Dermatologic toxicities are one of the most common irAEs from immunotherapy, which include maculopapular rash, pruritus, and psoriasiform and lichenoid eruptions [55,56]. About 30% to 40% of patients taking PD-1/PD-L1 inhibitors and 50% of patients taking CTLA-4 inhibitors experience dematologic irAEs [57]. After the initial dose of an immune checkpoint inhibitor, a maculopapular rash appears within the first six weeks, indicating there are cutaneous immune-related side effects. This rash can be managed with the use of topical corticosteroids for a mild to moderate rash, systemic corticosteroids for a severe rash, and immunotherapy treatment cessation for those with a potentially life-threatening rash such as Stevens-Johnson syndrome [55].

Another common type of irAEs are the gastrointestinal (GI) disorders that involve symptoms such as diarrhea and colitis. Up to 30% of patients receiving CTLA-4 inhibitors experience gastrointestinal-related AEs, and the percentage is even higher for patients receiving combination therapy, at 44% [58]. However, GI side effects are usually short-lived, about six weeks, and patients rarely suffer from ileal perforation. Symptomatic treatment alongside an adequate dietary adjustment to prevent dehydration is necessary for patients with grade 1 GI disorders, whereas those with grade 2 and colitis can be treated with oral or IV corticosteroids [58]. Hepatotoxicity induced by immune checkpoint inhibitors, on the other hand, is rather rare compared to GI AEs, but hepatitis still remains as part of the irAES. As hepatitis is usually asymptomatic, liver function tests are necessary for all patients before each treatment cycle, and once or twice a week if the aspartate aminotransferase (AST) and alanine aminotransferase (ALT) are elevated [58,59]. Patients with grade 1 hepatitis can still proceed with immune checkpoint inhibitors, provided that they are monitored closely, and treatment should be ceased in those with grade 3 or higher liver disorders until it subsides to grade 1 [58].

Additionally, inflammation of the myocardium and pericardium from the use of immune checkpoint inhibitors is thought to be caused by the existence of T-cell receptor sequences that are identical in cardiac muscle and tumors [60]. Like immune checkpoint inhibitors, CAR T-cell therapy has a similar cause of the cardiotoxicity mechanism of action, whereby the cardiac tissues and tumor cells share the same common antigens [60]. Nevertheless, treatment of cardiotoxicity is possible by managing the overactive T-cell response with therapies that are used to suppress the immune system. However, before initiation of treatment, how persistent the symptoms are must be considered, if the immunosuppressive therapy needs to be ongoing and if there are any life-threatening side effects [61].

In addition to the irAEs, endocrine-related irAEs include acute hypophysitis and thyroid disease, with hypophysitis being diagnosed two to five times more often in men of more than 60 years of age compared to women [62]. Patients receiving CTLA-4 inhibitors have a higher risk of developing hypophysitis, while those receiving PD-1/PD-L1 inhibitors possess a higher risk of primary thyroid dysfunction and, rarely, type 1 diabetes mellitus, central diabetic insipidus, and hypoparathyroidism [62]. Rarely, other immunotherapies such as oncolytic viruses, adoptive T-cell transfer, and cancer vaccines lead to thyroid dysfunctions [63]. Nevertheless, hormone replacement therapy is an effective treatment strategy in treating irAEs, if the patient has not previously experienced higher grades of irAEs’ toxicities.

Pulmonary irAEs derived from immunotherapy include interstitial lung disease and concomitant pneumonitis. Even though pulmonary toxicity is not the most common of AEs, it is nonetheless important, since it can be fatal [64]. Pneumonitis, the most common irAEs of the pulmonary system and the most common irAE-related cause of death, usually requires patients to discontinue immune checkpoint inhibitor therapy [65]. In most cases, immunotherapy is discontinued, and most patients are initiated with a low dose of corticosteroids accompanied by follow-up [64,66]. Restarting immunotherapy is possible if the patient recovers well without any complications.

Lastly, a significant number of irAEs have been recorded with CAR T-cell therapy, and the AEs include cytokine release syndrome (CRS), B-cell aplasia, anemia, thrombocytopenia, hypogammaglobulinemia, and neurological toxicities such as CAR T-cell related encephalopathy syndrome (CRES) [65,67,68]. CRS is clinically similar to sepsis and is driven by a significant release of pro-inflammatory cytokines. About 90% of patients on CAR T-cell treatment will experience CRS, with 50% requiring critical care and vasopressors and ventilation [65,68]. The start of the CRS symptoms usually occurs one to five days after CAR T-cell infusion, but it also varies depending on the agent and how severe the activation of the patient’s immune cells is [65]. Additionally, greater symptoms may be present in patients with large tumor masses. CRS management involves symptomatic treatment and cytokine inhibition, depending on the patient’s signs, symptoms, and hemodynamic status, as some might need IV fluids, vasopressors, and broad-spectrum antibiotics, when there is a possibility of sepsis [65]. Tocilizumab is effective in treating severe CRS, whereas corticosteroids are also considered but are often only used in combination with oncology consolation [65]. Otherwise, corticosteroids are often avoided, as they may have a negative impact on the antitumor effects. Meanwhile, tocilizumab in CRES is ineffective because it does not cross the blood–brain barrier, but anakinra, an IL-1 receptor antagonist, may help treat CRES [65]. IV corticosteroid dexamethasone is used to treat patients with severe neurologic symptoms, as it can cross the blood–brain barrier [65].

Cancer patients’ quality of life (QOL) is essential, as it affects how well their treatments work [69]. As cancer treatment continues to become more precise and focused over the years, cancer patients will be able to receive even more improved treatment outcomes with minimal adverse effects. According to Ramirez et al. (2018), immunotherapy produces a higher quality of life than the chemotherapy regimens used to treat various types of cancer. The incidences of grade 3 and higher adverse events with immunotherapy are lower compared to chemotherapy, meaning it can be considered to be safer than chemotherapy [70]. However, there are still patients that experience a significant amount of therapy-related adverse effects due to their treatment regimen, despite attempts to improve the QOL [70]. Hence, besides improving the survival rate, optimizing a patient’s QOL is crucial to reduce disease-related symptoms and therapy-related side effects.

## 5. Limitations

The sample size included in the clinical trials was small, as the studies were performed in small settings. Some of the studies did not provide any information on the median overall survival and progression free survival rates, as they were not assessed for the primary or secondary endpoints of the clinical trials, or there were not enough sufficient data to calculate the results, leading to limitations for the evaluation of the overall efficacy of the results.

## 6. Conclusions

In summary, more data are needed in order to comprehensively evaluate the overall efficacy of immunotherapy in cancer patients. Researchers who are designing new immunotherapy studies should ensure a larger group of patients’ recruitment. Nevertheless, despite the sample size, the results indicate the effectiveness of the immunotherapy used in the treatment of cancer patients, in prolonging their life span. In addition, immunotherapy is considered as a secondary alternative treatment option, when the primary standard treatment cannot be performed on some patients, such as the elderly. Overall, with the increasing rate of the aging population, immunotherapy offers a promising approach in the overall treatment of cancer, as a stand-alone treatment or in combination with other conventional cancer treatments.

## Figures and Tables

**Table 1:** The main characteristics and results of the studies are included in the systematic review.

Type of Cancer		Study Phase	Treatment Groups	Number of Patients	Mean Age, Years	Median Overall Survival, Months (95% CI); *p*-Value	Median Progression-Free Survival (95% CI); *p*-Value
Gastric Cancer	Wang et al. (2019) [10]	1b/2	A: Toripalimab B: Toripalimab plus XELOX (oxaliplatin, capecitabine)	A: 58 B: 18	A: 59.5 (52.0–66.0) B: 58.5 (48.0–69.0)	A: 4.8 months (N/A); *p* = N/A B: N/A	A: 1.9 months (N/A); *p* = N/A B: 5.8 months (N/A); *p* = N/A
Bladder Cancer	Shore et al. (2017) [11]	2	Low dose (LD) intravesical rAd-IFNα/Syn3 vs. high dose (HD) rAd-IFNα/Syn3	LD: 22 HD: 21	70.5 (64.5–77.5)	6.5 months (3.52–12.78)	LD: 3.52 months (3.02–12.78) HD: 11.73 months (5.88–N/A)
Non-Small Cell Lung Cancer	Ding et al. (2016) [12]	1b/2	Cytokine-induced killer (CIT group) vs. no treatment (control group)	49	CIT group: 63 (54–79) Control group: 57 (36–74)	CIT group: 13.3 months Control group: 8.2 months (N/A); *p* = 0.044	CIT group: 5 months Control group: 3.1 months (N/A); *p* = 0.020
	Cho et al. (2021) [13]	1	Quavonlimab plus pembrolizumab	40	66 (40–80)	11.0 months (5.9, 15.5); *p* = N/A	2.0 months (1.9, 3.9); *p* = N/A
	Planchard et al. (2020) [14]	3	A: Durvalumab vs. SoC B: Durvalumab plus tremelimumab (D + T) vs. SoC	A: 126 B: 469	A: Durvalumab 63.5 (35–79), SoC 62.0 (41–81) B: D + T 62.5 (26–81), SoC 65.0 (42–83)	A: Durvalumab 11.7 months (8.2, 17.4); *p* = 0, SoC 6.8 months (4.9, 10.2); *p* = 0 B: D + T 11.5 months (8.7, 14.1); *p* = 0, SoC 8.7 months (6.5, 11.7); *p* = 0	A: Durvalumab 3.8 months (1.9, 5.6); *p* = 0, SoC 2.2 months (1.9, 3.7); *p* = 0 B: D + T 9.1 months (6.6, 12.3); *p* = 0, SoC 3.5 months (1.9, 3.9); *p* = 0
	Hui et al. (2017) [15]	1	Pembrolizumab monotherapy	101	68.0 (N/A)	22.1 months (17.1–27.2); *p* = N/A	6.2 months (4.1, 8.6); *p* = N/A
	Spigel et al. (2018) [16]	2	Atezolizumab use in: Cohort 1: no previous treatment Cohort 2: prior platinum-based chemotherapy Cohort 3: prior platinum-based chemotherapy in brain metastases	Cohort 1: 31 Cohort 2: 93 Cohort 3: 13	1: 68 (42–85) 2: 65 (44–85) 3: 65 (52–74)	Cohort 1: 14.4 months (12.8, 22.1); *p* = N/A Cohort 2: 9.3 months (5.8, 17.6); *p* = N/A Cohort 3: 6.8 months (3.2, 19.4); *p* = N/A	Cohort 1: 4.5 months (3.3–8.3); *p* = N/A Cohort 2: 2.7 months (1.5–3.4); *p* = N/A Cohort 3: 2.5 months (1.2–4.2); *p* = N/A
Breast Cancer	Mittendorf et al. (2014) [17]	1/2	Vaccinated group (VC) E75 plus granulocyte-macrophage colony-stimulating factor (GM-CSF) vs. control group (CG) no treatment	187	VC: 57 (28–78) CG: 53 (32–83)	N/A	VC: 89.7% CG: 80.2% (N/A); *p* = 0.8
	Schmid et al. (2020) [18]	1b	Pembrolizumab plus chemotherapy	60	48.5 (26–71)	98% (90–100%); *p* = N/A	98% (90–100%); *p* = N/A
	Chumsri et al. (2019) [19]	3	Adjuvant chemotherapy plus trastuzumab vs. chemotherapy	3177	49.0 (23.0–80.0)	N/A	81.39% (78.54%–84.34%); *p* = N/A
Ovarian and Breast Cancer	Antonilli et al. (2016) [20]	1/2	Triple peptide vaccination	14	53.0 (42–70)	N/A	N/A
Glioblastoma	Liau et al. (2018) [21]	3	Temozolomide plus autologous tumor lysate-pulsed dendritic cell vaccine or Temozolomide plus placebo	331	56.0 (19–73)	23.1 (21.2-25.4)	N/A
Mesothelioma	Janssen et al. (2018) [22]	2	Nivolumab monotherapy	34	67.0 (50–81)	11.8 months (9.7–15.7); *p* = N/A	2.6 months (2.23–5.49); *p* = N/A
Cervical Cancer	Rischin et al. (2020) [23]	1	A: Cemiplimab monotherapy B: Cemiplimab plus hypofractionated radiation therapy (hfRT).	A: 10 B: 10	A: 55.0 (31.0–76.0) B: 51.5 (29.0–65.0)	A: 10.3 months (2.1–N/A); *p* = N/A B: 8.0 months (1.7–N/A); *p* = N/A	A: 1.9 months (1.0–9.0); *p* = N/A B: 3.6 months (0.6–5.7); *p* = N/A
	Harper et al. (2019) [24]	2b	A: Tipapkinogen Sovacivec vaccine B: placebo	206	A: 30.1 (18–60) B: 29.8 (19–50)	N/A	N/A
	Santin et al. (2020) [25]	2	Nivolumab monotherapy	26	45.0 (20–79)	14.5 months (8.3–26.8); *p* = N/A	3.5 months (1.9–5.1); *p* = N/A
Sarcoma	Ahmed et al. (2015) [26]	1/2	Human Epidermal Growth Factor Receptor 2 (HER2)—Specific Chimeric Antigen Receptor-Modified T Cells	19	17.0 (7.7–29.6)	10.3 months (5.1, 29.1); *p* = N/A	N/A
	Miwa et al. (2017) [27]	1/2	Dendritic cells pulsed with autologous tumor lysate	37	37.8 (8–65)	2.9% (N/A); *p* = N/A	42.3% (N/A); *p* = N/A
Head and Neck Squamous Cell Carcinoma	Ferris et al. (2020) [28]	3	A: Durvalumab vs. Soc B: Durvalumab plus tremelimumab vs. SoC	736	60.0 (N/A)	A: 7.6 months (6.1–9.8); *p* = 0.20 B: 6.5 months (5.5–8.2); *p* = 0.76	A: 2.1 months (1.9–3.0); *p* = N/A B: 2.0 months (1.9–2.3); *p* = N/A
	Saba et al. (2019) [29]	3	A: Nivolumab vs. SoC in < 65 years old patients. B: Nivolumab vs. SoC in ≥ 65-year-old patients	361	48.5 (26–71)	A: 8.2 months vs. 4.9 months (0.47–0.84); *p* = N/A B: 6.9 months vs. 6.0 months (0.51–1.12); *p* = N/A	A: 2.0 months vs. 2.7 months (0.71–1.30); *p* = N/A B: 2.1 months vs. 2.0 months (0.49–1.11); *p* = N/A
	Zandberg et al. (2019) [30]	2	Durvalumab monotherapy	112	60.0 (24.0–84.0)	7.1 months (1.9–5.6); *p* = N/A	2.1 months (1.9–3.7); *p* = N/A
Esophageal Squamous Cell Carcinoma	Zhang et al. (2020) [31]	2	Camrelizumab plus apatinib and chemotherapy	30	61.5 (43–70)	19.43 months (9.93–N/A); *p* = N/A	6.85 months (4.46–14.20); *p* = N/A
Prostate Cancer	Hansen et al. (2018) [32]	1b	Pembrolizumab monotherapy	245	65.0 (46–83)	7.9 months (6.5–N/A); *p* = N/A	3.5 months (1.7–6.5); *p* = N/A
	Schuhmacher et al. (2020) [33]	1/2	Ras homolog gene family member C vaccination	22	66.0 (54–77)	N/A	N/A
Melanoma	Garbe et al. (2008) [34]	3	Adjuvant interferon α2a with or without dacarbazine vs. surgery	444	N/A	59.0% vs. 42.0% (N/A); *p* = 0.0045	39.0% vs. 27.0% (N/A); *p* = 0.018
	Namikawa et al. (2018) [35]	2	Nivolumab plus ipilimumab	30	58.5 (31–81)	N/A	N/A
	Hemstock et al. (2020) [36]	3	Nivolumab vs. placebo	928	N/A	N/A	N/A
Leukemia	Anguille et al. (2017) [37]	2	Adjuvant dendritic cell vaccination	30	60.0 (30–79)	41.8 months (N/A); *p* = N/A	N/A
	Kreitman et al. (2021) [38]	3	Moxetummomab pasudotox	80	60	N/A	41.5 months (29.5, N/A); *p* = N/A
Lymphoma	Wang et al. (2020) [39]	2	KTE-X19 CAR T-Cell therapy	60	65.0 (38–79)	N/A	N/A
	Maruyama et al. (2017) [40]	2	Nivolumab	17	63.0 (29–83)	N/A	N/A
	Fan et al. (2014) [41]	1/2	A: Decitabine B: Decitabine plus chemotherapy C: Decitabine plus cytokine induced killer cells	32	58.8 (28–84)	N/A	A: 2.5 months (1–12); *p* = N/A B: 4 months (1–7); *p* = N/A C: 8 months (4–10); *p* = N/A
Malignant Ascites	Heiss et al. (2010) [42]	2/3	A: Paracentesis plus catumaxomab B: Paracentesis alone	258	N/A	A: 72 days (61–96); *p* = N/A B: 68 days (49–81); *p* = N/A	A: 46 days (35–53); *p* = N/A B: 11 days (9–16); *p* = N/A
	Burges et al. (2007) [43]	1/2	Catumaxomab	23	61.7 (42–80)	N/A	N/A

N/A = not available, SoC = standard of care.

**Table 2:** (**A**) Risk of bias for non-randomized controlled trials. (**B**) Risk of bias for randomized controlled trials.

**(A)**									
**Study**		**Pre-Intervention**		**At Intervention**	**Post Intervention**				**Overall Risk of Bias**
**First Author**	**Year**	**Bias Due to Confounding**	**Bias in Selection of Participants into the Study**	**Bias in Classification of Interventions**	**Bias Due to Deviations from Intended Interventions**	**Bias Due to Missing Data**	**Bias in Measurement of Outcomes**	**Bias in Selection of the Reported Result**	**Low, Moderate, Serious, Critical**
Wang et al. [10]	2019	Low	Low	Low	Low	Low	Moderate	Low	Moderate
Ding et al. [12]	2016	Low	Low	Low	Low	Low	Moderate	Low	Moderate
Rischin et al. [23]	2020	Low	Low	Low	Low	Low	Moderate	Low	Moderate
Mittendorf et al. [17]	2014	Low	Low	Low	Moderate	Low	Moderate	Low	Moderate
Cho et al. [13]	2021	Low	Low	Low	Low	Low	Moderate	Low	Moderate
Janssen et al. [22]	2018	Low	Low	Low	Low	Low	Moderate	Low	Moderate
Hansen et al. [32]	2018	Moderate	Low	Low	Low	Low	Moderate	Low	Moderate
Spigel et al. [16]	2018	Moderate	Low	Low	Low	Low	Moderate	Low	Moderate
Zandberg et al. [30]	2019	Low	Low	Low	Low	Low	Moderate	Low	Moderate
Anguille et al. [37]	2017	Low	Low	Low	Low	Low	Moderate	Low	Moderate
Namikawa et al. [35]	2018	Low	Low	Low	Low	Low	Moderate	Low	Moderate
Wang et al. [39]	2020	Low	Low	Low	Low	Low	Moderate	Low	Moderate
Santin et al. [25]	2020	Low	Low	Low	Low	Low	Moderate	Low	Moderate
Zhang et al. [31]	2020	Low	Low	Low	Low	Low	Moderate	Low	Moderate
Ahmed et al. [26]	2015	Low	Low	Low	Low	Low	Moderate	Low	Moderate
Schuhmacher et al. [33]	2020	Low	Low	Low	Low	Low	Moderate	Low	Moderate
Antonilli et al. [20]	2016	Low	Low	Low	Low	Low	Moderate	Low	Moderate
Maruyama et al. [40]	2017	Low	Low	Low	Low	Low	Moderate	Low	Moderate
Fan et al. [41]	2014	Low	Low	Low	Low	Low	Moderate	Low	Moderate
Burges et al. [43]	2007	Low	Low	Low	Low	Low	Moderate	Low	Low
Miwa et al. [27]	2017	Low	Low	Low	Low	Low	Moderate	Low	Moderate
Kreitman et al. [38]	2021	Low	Low	Low	Low	Low	Moderate	Low	Moderate
**(B)**									
**Study**		**Pre-Intervention**			**Post Intervention**				**Overall Risk of Bias**
**First Author**	**Year**	**Bias Arising from the Randomization Process**			**Bias Due to Deviations from Intended Interventions**	**Bias Due to Missing Outcome Data**	**Bias in Measurement of the Outcome**	**Bias in Selection of the Reported Result**	**Low, Some Concerns, High Risk of Bias**
Hui et al. [15]	2017	Some concerns			Some concerns	Low	Low	Low	Some concerns
Schmid et al. [18]	2020	Low			Some concerns	Low	Low	Low	Some concerns
Harper et al. [24]	2019	Some concerns			Low	Low	Low	Low	Some concerns
Ferris et al. [28]	2020	Some concerns			Some concerns	Low	Low	Low	Some concerns
Saba et al. [29]	2019	Some concerns			Some concerns	Low	Low	Low	Some concerns
Garbe et al. [34]	2008	Some concerns			Some concerns	Low	Low	Low	Some concerns
Heiss et al. [42]	2010	Some concerns			Some concerns	Low	Low	Low	Some concerns
Chumsri et al. [19]	2019	Some concerns			Some concerns	Low	Low	Low	Some concerns
Shore et al. [11]	2017	Low			Some concerns	Low	Low	Low	Some concerns
Liau et al. [21]	2018	Low			Some concerns	Low	Low	Low	Some concerns
Planchard et al. [14]	2020	Some concerns			Some concerns	Low	Low	Low	Some concerns
Hemstock et al. [36]	2020	Some concerns			Low	Low	Low	Low	Some concerns

## References

[1] Sung H., Ferlay J., Siegel R.L., Laversanne M., Soerjomataram I., Jemal A., Bray F. (2021). Global Cancer Statistics 2020: GLOBOCAN Estimates of Incidence and Mortality Worldwide for 36 Cancers in 185 Countries. CA Cancer J. Clin..

[2] Xiaomei M., Herbert Y. (2006). Cancer Issue: Global Burden of Cancer. Yale J. Biol. Med..

[3] Arruebo M., Vilaboa N., Sáez-Gutierrez B., Lambea J., Tres A., Valladares M., González-Fernández Á. (2011). Assessment of the Evolution of Cancer Treatment Therapies. Cancers.

[4] Whitaker K. (2019). Earlier diagnosis: The importance of cancer symptoms. Lancet Oncol..

[5] Borghaei H., Smith M.R., Campbell K.S. (2009). Immunotherapy of cancer. Eur. J. Pharmacol..

[6] Advances in Cancer Immunology and Cancer Immunotherapy—PubMed. https://pubmed.ncbi.nlm.nih.gov/27011048/.

[7] Mellman I., Coukos G., Dranoff G. (2011). Cancer immunotherapy comes of age. Nature.

[8] Sterne J.A.C., Hernán M.A., Reeves B.C., Savović J., Berkman N.D., Viswanathan M., Henry D., Altman D.G., Ansari M.T., Boutron I. (2016). ROBINS-I: A tool for assessing risk of bias in non-randomised studies of interventions. BMJ.

[9] Cochrane Handbook for Systematic Reviews and Interventions. http://training.cochrane.org/handbook.

[10] Wang F.H., Wei X., Xu N., Shen L., Dai G., Yuan X., Chen Y., Yang S., Shi J., Hu X. (2019). Safety, efficacy and tumor mutational burden as a biomarker of overall survival benefit in chemo-refractory gastric cancer treated with toripalimab, a PD-1 antibody in phase Ib/II clinical trial NCT02915432. Ann. Oncol..

[11] Shore N.D., Boorjian S.A., Canter D.J., Ogan K., Karsh L.I., Downs T.M., Gomella L.G., Kamat A.M., Lotan Y., Svatek R.S. (2017). Intravesical rAd–IFNα/Syn3 for Patients with High-Grade, Bacillus Calmette-Guerin–Refractory or Relapsed Non–Muscle-Invasive Bladder Cancer: A Phase II Randomized Study. J. Clin. Oncol..

[12] Ding X., Cao H., Chen X., Zhao Y., Jin H., Niu C., Ma K., Liu Z., Chen J., Wang X. (2016). Cellular immunotherapy as maintenance therapy prolongs the survival of the patients with small cell lung cancer in extensive stage. J. Cell. Immunother..

[13] Cho B.C., Yoh K., Perets R., Nagrial A., Spigel D.R., Gutierrez M., Kim D.-W., Kotasek D., Rasco D., Niu J. (2021). Anti–cytotoxic T-lymphocyte–associated antigen-4 monoclonal antibody quavonlimab in combination with pembrolizumab: Safety and efficacy from a phase I study in previously treated extensive-stage small cell lung cancer. Lung Cancer.

[14] Planchard D., Reinmuth N., Orlov S., Fischer J., Sugawara S., Mandziuk S., Marquez-Medina D., Novello S., Takeda Y., Soo R. (2020). ARCTIC: Durvalumab with or without tremelimumab as third-line or later treatment of metastatic non-small-cell lung cancer. Ann. Oncol..

[15] Hui R., Garon E.B., Goldman J.W., Leighl N.B., Hellmann M.D., Patnaik A., Gandhi L., Eder J.P., Ahn M.-J., Horn L. (2017). Pembrolizumab as first-line therapy for patients with PD-L1-positive advanced non-small cell lung cancer: A phase 1 trial. Ann. Oncol..

[16] Spigel D.R., Chaft J.E., Gettinger S., Chao B.H., Dirix L., Schmid P., Chow L.Q., Hicks R.J., Leon L., Fredrickson J. (2018). FIR: Efficacy, Safety, and Biomarker Analysis of a Phase II Open-Label Study of Atezolizumab in PD-L1–Selected Patients With NSCLC. J. Thorac. Oncol..

[17] Mittendorf E., Clifton G., Holmes J., Schneble E., van Echo D., Ponniah S., Peoples G. (2014). Final report of the phase I/II clinical trial of the E75 (nelipepimut-S) vaccine with booster inoculations to prevent disease recurrence in high-risk breast cancer patients. Ann. Oncol..

[18] Schmid P., Salgado R., Park Y., Muñoz-Couselo E., Kim S., Sohn J., Im S.-A., Foukakis T., Kuemmel S., Dent R. (2020). Pembrolizumab plus chemotherapy as neoadjuvant treatment of high-risk, early-stage triple-negative breast cancer: Results from the phase 1b open-label, multicohort KEYNOTE-173 study. Ann. Oncol..

[19] Chumsri S., Li Z., Serie D.J., Mashadi-Hossein A., Colon-Otero G., Song N., Pogue-Geile K.L., Gavin P., Paik S., Moreno-Aspitia A. (2019). Incidence of Late Relapses in Patients with HER2-Positive Breast Cancer Receiving Adjuvant Trastuzumab: Combined Analysis of NCCTG N9831 (Alliance) and NRG Oncology/NSABP B-31. J. Clin. Oncol..

[20] Antonilli M., Rahimi H., Visconti V., Napoletano C., Ruscito I., Zizzari I.G., Caponnetto S., Barchiesi G., Iadarola R., Pierelli L. (2016). Triple peptide vaccination as consolidation treatment in women affected by ovarian and breast cancer: Clinical and immunological data of a phase I/II clinical trial. Int. J. Oncol..

[21] Liau L.M., Ashkan K., Tran D.D., Campian J.L., Trusheim J.E., Cobbs C.S., Heth J.A., Salacz M., Taylor S., D’Andre S.D. (2018). First results on survival from a large Phase 3 clinical trial of an autologous dendritic cell vaccine in newly diagnosed glioblastoma. J. Transl. Med..

[22] Quispel-Janssen J., van der Noort V., de Vries J.F., Zimmerman M., Lalezari F., Thunnissen E., Monkhorst K., Schouten R., Schunselaar L., Disselhorst M. (2018). Programmed Death 1 Blockade with Nivolumab in Patients With Recurrent Malignant Pleural Mesothelioma. J. Thorac. Oncol..

[23] Rischin D., Gil-Martin M., González-Martin A., Braña I., Hou J.Y., Cho D., Falchook G.S., Formenti S., Jabbour S., Moore K. (2020). PD-1 blockade in recurrent or metastatic cervical cancer: Data from cemiplimab phase I expansion cohorts and characterization of PD-L1 expression in cervical cancer. Gynecol. Oncol..

[24] Harper D.M., Nieminen P., Donders G., Einstein M.H., Garcia F., Huh W.K., Stoler M.H., Glavini K., Attley G., Limacher J.-M. (2019). The efficacy and safety of Tipapkinogen Sovacivec therapeutic HPV vaccine in cervical intraepithelial neoplasia grades 2 and 3: Randomized controlled phase II trial with 2.5 years of follow-up. Gynecol. Oncol..

[25] Santin A.D., Deng W., Frumovitz M., Buza N., Bellone S., Huh W., Khleif S., Lankes H.A., Ratner E.S., O’Cearbhaill R.E. (2020). Phase II evaluation of nivolumab in the treatment of persistent or recurrent cervical cancer (NCT02257528/NRG-GY002). Gynecol. Oncol..

[26] Ahmed N., Brawley V.S., Hegde M., Robertson C., Ghazi A., Gerken C., Liu E., Dakhova O., Ashoori A., Corder A. (2015). Human Epidermal Growth Factor Receptor 2 (HER2)–Specific Chimeric Antigen Receptor–Modified T Cells for the Immunotherapy of HER2-Positive Sarcoma. J. Clin. Oncol..

[27] Miwa S., Nishida H., Tanzawa Y., Takeuchi A., Hayashi K., Yamamoto N., Mizukoshi E., Nakamoto Y., Kaneko S., Tsuchiya H. (2017). Phase 1/2 study of immunotherapy with dendritic cells pulsed with autologous tumor lysate in patients with refractory bone and soft tissue sarcoma. Cancer.

[28] Ferris R., Haddad R., Even C., Tahara M., Dvorkin M., Ciuleanu T., Clement P., Mesia R., Kutukova S., Zholudeva L. (2020). Durvalumab with or without tremelimumab in patients with recurrent or metastatic head and neck squamous cell carcinoma: EAGLE, a randomized, open-label phase III study. Ann. Oncol..

[29] Saba N.F., Blumenschein G., Guigay J., Licitra L., Fayette J., Harrington K.J., Kiyota N., Gillison M.L., Ferris R.L., Jayaprakash V. (2019). Nivolumab versus investigator’s choice in patients with recurrent or metastatic squamous cell carcinoma of the head and neck: Efficacy and safety in CheckMate 141 by age. Oral Oncol..

[30] Zandberg D.P., Algazi A.P., Jimeno A., Good J.S., Fayette J., Bouganim N., Ready N.E., Clement P.M., Even C., Jang R.W. (2019). Durvalumab for recurrent or metastatic head and neck squamous cell carcinoma: Results from a single-arm, phase II study in patients with ≥25% tumour cell PD-L1 expression who have progressed on platinum-based chemotherapy. Eur. J. Cancer.

[31] Zhang B., Qi L., Wang X., Xu J., Liu Y., Mu L., Wang X., Bai L., Huang J. (2020). Phase II clinical trial using camrelizumab combined with apatinib and chemotherapy as the first-line treatment of advanced esophageal squamous cell carcinoma. Cancer Commun..

[32] Hansen A.R., Massard C., Ott P.A., Haas N.B., Lopez J.S., Ejadi S., Wallmark J.M., Keam B., Delord J.-P., Aggarwal R. (2018). Pembrolizumab for advanced prostate adenocarcinoma: Findings of the KEYNOTE-028 study. Ann. Oncol..

[33] Schuhmacher J., Heidu S., Balchen T., Richardson J.R., Schmeltz C., Sonne J., Schweiker J., Rammensee H.-G., Straten P.T., Røder M.A. (2020). Vaccination against RhoC induces long-lasting immune responses in patients with prostate cancer: Results from a phase I/II clinical trial. J. Immunother. Cancer.

[34] Garbe C., Radny P., Linse R., Dummer R., Gutzmer R., Ulrich J., Stadler R., Weichenthal M., Eigentler T., Ellwanger U. (2008). Adjuvant low-dose interferon α2a with or without dacarbazine compared with surgery alone: A prospective-randomized phase III DeCOG trial in melanoma patients with regional lymph node metastasis. Ann. Oncol..

[35] Namikawa K., Kiyohara Y., Takenouchi T., Uhara H., Uchi H., Yoshikawa S., Takatsuka S., Koga H., Wada N., Minami H. (2018). Efficacy and safety of nivolumab in combination with ipilimumab in Japanese patients with advanced melanoma: An open-label, single-arm, multicentre phase II study. Eur. J. Cancer.

[36] Hemstock M., Amadi A., Kupas K., Roskell N., Kotapati S., Gooden K., Middleton M.R., Schadendorf D. (2020). Indirect treatment comparison of nivolumab versus placebo for the adjuvant treatment of melanoma. Eur. J. Cancer.

[37] Anguille S., Van de Velde A.L., Smits E.L., Van Tendeloo V.F., Juliusson G., Cools N., Nijs G., Stein B., Lion E., Van Driessche A. (2017). Dendritic cell vaccination as postremission treatment to prevent or delay relapse in acute myeloid leukemia. Blood.

[38] Kreitman R.J., Dearden C., Zinzani P.L., Delgado J., Robak T., le Coutre P.D., Gjertsen B.T., Troussard X., Roboz G.J., Karlin L. (2021). Moxetumomab pasudotox in heavily pre-treated patients with relapsed/refractory hairy cell leukemia (HCL): Long-term follow-up from the pivotal trial. J. Hematol. Oncol..

[39] Wang M., Munoz J., Goy A., Locke F.L., Jacobson C.A., Hill B.T., Timmerman J.M., Holmes H., Jaglowski S., Flinn I.W. (2020). KTE-X19 CAR T-Cell Therapy in Relapsed or Refractory Mantle-Cell Lymphoma. N. Engl. J. Med..

[40] Maruyama D., Hatake K., Kinoshita T., Fukuhara N., Choi I., Taniwaki M., Ando K., Terui Y., Higuchi Y., Onishi Y. (2017). Multicenter phase II study of nivolumab in Japanese patients with relapsed or refractory classical Hodgkin lymphoma. Cancer Sci..

[41] Fan H., Lu X., Wang X., Liu Y., Guo B., Zhang Y., Zhang W., Nie J., Feng K., Chen M. (2014). Low-Dose Decitabine-Based Chemoimmunotherapy for Patients with Refractory Advanced Solid Tumors: A Phase I/II Report. J. Immunol. Res..

[42] Heiss M.M., Murawa P., Koralewski P., Kutarska E., Kolesnik O.O., Ivanchenko V.V., Dudnichenko A.S., Aleknaviciene B., Razbadauskas A., Gore M. (2010). The trifunctional antibody catumaxomab for the treatment of malignant ascites due to epithelial cancer: Results of a prospective randomized phase II/III trial. Int. J. Cancer.

[43] Burges A., Wimberger P., Kümper C., Gorbounova V., Sommer H., Schmalfeldt B., Pfisterer J., Lichinitser M., Makhson A., Moiseyenko V. (2007). Effective Relief of Malignant Ascites in Patients with Advanced Ovarian Cancer by a Trifunctional Anti-EpCAM × Anti-CD3 Antibody: A Phase I/II Study. Clin. Cancer Res..

[44] Desai K., McManus J.M., Sharifi N. (2021). Hormonal Therapy for Prostate Cancer. Endocr. Rev..

[45] Daniel M., Keefe F.J., Lyna P., Peterson B., Garst J., Kelley M., Bepler G., Bastian L.A. (2009). Persistent Smoking After a Diagnosis of Lung Cancer Is Associated with Higher Reported Pain Levels. J. Pain.

[46] Yanyan H., Dandan L., Lianhong L. (2020). PD-1/PD-L1 Pathway: Current Researches in Cancer. Am. J. Cancer Res..

[47] Benjamin D.J., Xu A., Lythgoe M.P., Prasad V. (2022). Cancer Drug Approvals That Displaced Existing Standard-of-Care Therapies, 2016-2021. JAMA Netw. Open.

[48] Kichloo A., Albosta M., Dahiya D., Guidi J.C., Aljadah M., Singh J., Shaka H., Wani F., Kumar A., Lekkala M. (2021). Systemic adverse effects and toxicities associated with immunotherapy: A review. World J. Clin. Oncol..

[49] Migden M.R., Rischin D., Schmults C.D., Guminski A., Hauschild A., Lewis K.D., Chung C.H., Hernandez-Aya L.F., Lim A.M., Chang A.L.S. (2018). PD-1 Blockade with Cemiplimab in Advanced Cutaneous Squamous-Cell Carcinoma. N. Engl. J. Med..

[50] Ahmad A., Uddin S., Steinhoff M. (2020). CAR-T Cell Therapies: An Overview of Clinical Studies Supporting Their Approved Use against Acute Lymphoblastic Leukemia and Large B-Cell Lymphomas. Int. J. Mol. Sci..

[51] Voelker R. (2020). Immunotherapy Is Now First-line Therapy for Some Colorectal Cancers. JAMA.

[52] Xiong A., Wang J., Zhou C. (2021). Immunotherapy in the First-Line Treatment of NSCLC: Current Status and Future Directions in China. Front. Oncol..

[53] Brown T.J., Mamtani R., Bange E.M. (2021). Immunotherapy Adverse Effects. JAMA Oncol..

[54] Barber F.D. (2019). Adverse Events of Oncologic Immunotherapy and Their Management. Asia-Pac. J. Oncol. Nurs..

[55] Geisler A.N., Phillips G.S., Barrios D.M., Wu J., Leung D.Y.M., Moy A.P., Kern J.A., Lacouture M.E. (2020). Immune checkpoint inhibitor–related dermatologic adverse events. J. Am. Acad. Dermatol..

[56] Si X., He C., Zhang L., Liu X., Li Y., Wang H., Guo X., Zhou J., Duan L., Wang M. (2019). Management of immune checkpoint inhibitor-related dermatologic adverse events. Thorac. Cancer.

[57] Apalla Z., Rapoport B., Sibaud V. (2021). Dermatologic immune-related adverse events: The toxicity spectrum and recommendations for management. Int. J. Women’s Dermatol..

[58] Liu Y.-H., Zang X.-Y., Wang J.-C., Huang S.-S., Xu J., Zhang P. (2019). Diagnosis and Management of Immune Related Adverse Events (irAEs) in Cancer Immunotherapy. Biomed. Pharmacother..

[59] Rajha E., Chaftari P., Kamal M., Maamari J., Chaftari C., Yeung S.-C.J. (2019). Gastrointestinal adverse events associated with immune checkpoint inhibitor therapy. Gastroenterol. Rep..

[60] Ganatra S., Parikh R., Neilan T.G. (2019). Cardiotoxicity of Immune Therapy. Cardiol. Clin..

[61] Ala C.K., Klein A.L., Moslehi J.J. (2019). Cancer Treatment-Associated Pericardial Disease: Epidemiology, Clinical Presentation, Diagnosis, and Management. Curr. Cardiol. Rep..

[62] Del Rivero J., Cordes L.M., Klubo-Gwiezdzinska J., Madan R.A., Nieman L.K., Gulley J.L. (2019). Endocrine-Related Adverse Events Related to Immune Checkpoint Inhibitors: Proposed Algorithms for Management. Oncologist.

[63] Chalan P., Di Dalmazi G., Pani F., De Remigis A., Corsello A., Caturegli P. (2017). Thyroid dysfunctions secondary to cancer immunotherapy. J. Endocrinol. Investig..

[64] Delaunay M., Prévot G., Collot S., Guilleminault L., Didier A., Mazières J. (2019). Management of pulmonary toxicity associated with immune checkpoint inhibitors. Eur. Respir. Rev..

[65] Long B.J., Brem E., Koyfman A. (2020). Oncologic Emergencies: Immune-Based Cancer Therapies and Complications. West. J. Emerg. Med..

[66] Chuzi S., Tavora F., Cruz M., Costa R., Chae Y.K., Carneiro A.B., Giles F.J. (2017). Clinical features, diagnostic challenges, and management strategies in checkpoint inhibitor-related pneumonitis. Cancer Manag. Res..

[67] Lipe D.N., Shafer S. (2021). CAR-T and checkpoint inhibitors: Toxicities and antidotes in the emergency department. Clin. Toxicol..

[68] Pallin D.J., Baugh C.W., Postow M.A., Caterino J.M., Erickson T.B., Lyman G.H. (2018). Immune-related Adverse Events in Cancer Patients. Acad. Emerg. Med..

[69] Sibeoni J., Picard C., Orri M., Labey M., Bousquet G., Verneuil L., Revah-Levy A. (2018). Patients’ quality of life during active cancer treatment: A qualitative study. BMC Cancer.

[70] Ramirez R.A., Lu J., Thomas K.E.H. (2018). Quality of life for non-small cell lung cancer patients in the age of immunotherapy. Transl. Lung Cancer Res..

